# Microbiota profiling in esophageal diseases: Novel insights into molecular staining and clinical outcomes

**DOI:** 10.1016/j.csbj.2023.12.026

**Published:** 2023-12-27

**Authors:** Alberto Barchi, Luca Massimino, Francesco Vito Mandarino, Edoardo Vespa, Emanuele Sinagra, Omar Almolla, Sandro Passaretti, Ernesto Fasulo, Tommaso Lorenzo Parigi, Stefania Cagliani, Salvatore Spanò, Federica Ungaro, Silvio Danese

**Affiliations:** a Gastroenterology and Digestive Endoscopy, IRCCS Ospedale San Raffaele, Milan, Italy; b Università Vita-Salute San Raffaele, Faculty of Medicine, Milan, Italy; c Gastroenterology & Endoscopy Unit, Fondazione Istituto G. Giglio, Cefalù, Italy

**Keywords:** Microbiota, Esophagus, Eosinophilic esophagitis, Achalasia, Cancer

## Abstract

Gut microbiota is recognized nowadays as one of the key players in the development of several gastro-intestinal diseases. The first studies focused mainly on healthy subjects with staining of main bacterial species via culture-based techniques. Subsequently, lots of studies tried to focus on principal esophageal disease enlarged the knowledge on esophageal microbial environment and its role in pathogenesis. Gastro Esophageal Reflux Disease (GERD), the most widespread esophageal condition, seems related to a certain degree of mucosal inflammation, via interleukin (IL) 8 potentially enhanced by bacterial components, lipopolysaccharide (LPS) above all. Gram^-^ bacteria, producing LPS), such as Campylobacter genus, have been found associated with GERD. Barrett esophagus (BE) seems characterized by a Gram^-^ and microaerophils-shaped microbiota. Esophageal cancer (EC) development leads to an overturn in the esophageal environment with the shift from an oral-like microbiome to a prevalently low-abundant and low-diverse Gram^-^-shaped microbiome. Although underinvestigated, also changes in the esophageal microbiome are associated with rare chronic inflammatory or neuropathic disease pathogenesis. The paucity of knowledge about the microbiota-driven mechanisms in esophageal disease pathogenesis is mainly due to the scarce sensitivity of sequencing technology and culture methods applied so far to study commensals in the esophagus. However, the recent advances in molecular techniques, especially with the advent of non-culture-based genomic sequencing tools and the implementation of multi-omics approaches, have revolutionized the microbiome field, with promises of implementing the current knowledge, discovering more mechanisms underneath, and giving insights into the development of novel therapies aimed to re-establish the microbial equilibrium for ameliorating esophageal diseases..

## 1 Introduction

The gastrointestinal barrier consists of a multilayer structure, mainly designated to absorb nutrients, regulate the homeostasis of the whole organism, and balance immunity against exogenous insults [1]. The commensal communities inhabiting the intestinal mucosa surface represent an essential layer and integral part of the gastrointestinal tract (GI), namely the gut microbiota, defined as a complex ecosystem of trillions of microorganisms, including archaea, bacteria, viruses, protists, and eukaryotes [2] that co-exist with predator-pray relationships, interacting with each other and with the host [3]. Gut microbiota gained interest from the scientific community only in the early 2000 s, when its role was established as key in modulating physiological functions and contributing to disease onset and progression [4]. While the microbiota in the lower tract of the intestine, namely the small and large bowel, has been the core of scientific interest over the last 20 years [5], [6], the resident microbial flora of the esophagus and its potential role in the pathogenesis of esophageal diseases have been neglected for years.

Pioneer studies on esophageal microbiota came out only in the early 80’s, when, by culture-dependent techniques, Mannell and colleagues identified *Streptococcus viridans*, *Haemophilus influenzae, Neisseria catarrhalis, Streptococcus group B, Streptococcus faecalis* and *Klebsiella pneumoniae* as commensals populating the esophageal surface in healthy subjects [7]. Gram^+^ bacterial prevalence was confirmed in 1998 by Gagliardi and colleagues analyzing esophageal microflora in patients suffering from dyspeptic symptoms [8]. The authors assessed a prevalence of *S. Viridans* and *K. Pneumoniae* both in the esophagus aspirate as well as in oropharynx samples, raising the question of whether the esophageal microbiome may be transiently affected by oral microbial species [9]. In this regard, Norder Grusell and colleagues, via culture-indipendent techniques, identified differences in particular species of microbes between esophageal and oral cavity samples, confirming the existence of a resident microbiota esophageal signature, with distinct characteristics from the oral one. Beyond these low-numbered distinct features, resident esophageal microbial species showed strong similarities to the oral ones, being *S. Viridans* and *H. Influenzae* were the most prevalent [10].

**Fig. 1:**
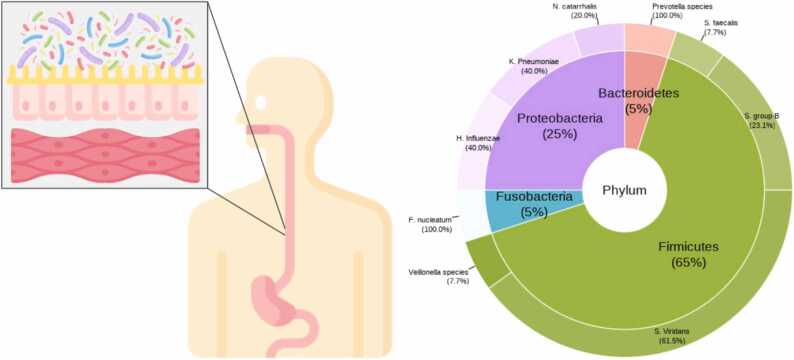
Main phyla, families, and species of esophageal microbiota in healthy individuals.

Through the 16S rRNA sequencing technique, other authors have described *Streptococcacae*, *Veilonellacae,* and *Prevotellacae* as the predominant families resident in the healthy esophagus [11], [12], [13] (Fig. 1). Esophageal microbiota has been also evaluated in pathological contexts, where, the diseased mucosa, as well as the pharmacological treatments or dietary habits [13], [14] can impact the equilibrium among the commensals, leading to dysbiosis. For example, in Gastrosophageal Reflux Disease (GERD) a prevalence of Gram^-^ bacteria has been found, with the potential etiopathogenetic role of lipopolysaccharide (LPS) in disease genesis [15]. The role of a Gram^-^ bacteria-shaped esophageal microbiota has been evaluated also as a co-factor in the carcinogenesis cascade, starting from early epithelial modifications, leading to Barrett Esophagus (BE) and ultimately to esophageal cancer (EC) [16].

In this context, technological advancements made by high-throughput microbial RNA and DNA sequencing techniques have revolutionized gut microbiota analysis, increasing the quantity and the quality of data, abating costs, and maximizing research efforts [17]. Interesting insights on relationships between esophageal microbiome and inflammatory or neurodegenerative esophageal conditions like Eosinophilic Esophagitis (EoE) and achalasia have been recently arisen [18]. Together with advanced sequencing techniques, computational biology and informatics knowledge has spread in recent years improving efforts and reducing time and costs in microbiota study and shaping [19].

The purpose of this narrative review is to summarize the current evidence on esophageal microbiome composition and its role during esophageal disease pathogenesis, and how multi-omics approaches together with bioinformatics innovations have revolutionized this field.

## 2 Advanced computational approaches for microbial profiling of esophageal tracts in health and disease

Esophageal microbiota refers to the dynamic community of microorganisms that inhabit the esophagus [20], influenced by several factors, including diet, age, and diseases [21], and proposed to harbor a role in several esophageal disorders, including eosinophilic esophagitis (EoE), gastroesophageal reflux disease (GERD), esophageal carcinomas and carcinogenesis, and achalasia [22]. However, the exact mechanisms by which microbiota contributes to these diseases are not completely understood [16].

In recent years, the use of molecular and computational advanced techniques to profile esophageal microbiota has gained increasing attention [23]. These techniques have enabled researchers to better understand the microbiota composition and diversity, as well as the relationships between the esophageal microbiota and the host [3]. The breakthrough was the advent of high-throughput sequencing technologies to profile microbial genomes, with *16S rRNA* gene sequencing [24] and internal transcribed spacer (ITS) region sequencing for bacterial and fungal species, respectively [25].

However, despite the relevance of these technologies, the main limitation was the small number of genes analyzed, with a higher risk of accumulating sequencing errors through the Polymerase Chain Reaction (PCR) [26]. Further limitations arise from the identical genome sequences between different microbial species, representing a challenge in discriminating which species is responsible for the transcription of that particular gene product [17].

Some of the aforementioned limitations are overcome by shotgun metagenomics having as outputs the whole genome of the microbial environment [27]. However, its limitation is the lack of a consensus in interpreting results, depending on the quality of reference databases used and the data assembly method performed [28].

Another recently established analysis is meta-transcriptomics (the analysis of the whole transcripts beyond the human organism), which has the potential to encode specific microbial gene products [29] and, coupled with the host’s transcriptomics, provides information about the possible impact of the microbiota on the host’s physiology [33].

Similarly, metabolomics and meta-proteomics, the analysis through mass spectrometry [30] of the proteins produced in the microbial environment and their specific metabolites with their interaction with the host has lately produced a comprehensive understanding of the biochemical processes underneath some human diseases [31].

**Fig. 2:**
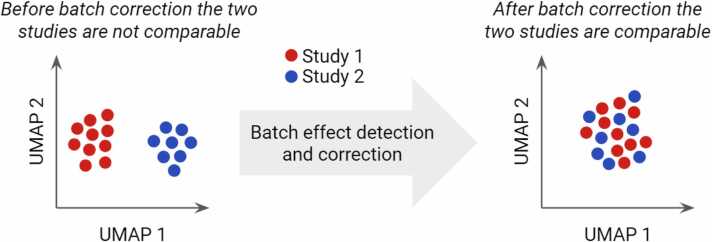
Batch correction analysis.

In this intricacy, novel tools for computational analysis are essential. Meta-transcriptomic methods are mainly performed using Illumina sequencing methodologies, such as HiSeq or NovaSeq with high throughput and fairly low costs [32]. A recently developed and widely used tool for gene expression analysis is certainly DESeq2 [33]. For the analysis of gene expression related to the metatranscriptome, the distinction and taxonomic classification of the microbial reads coming from the sequencing is crucial. Such taxonomic classification can be performed using tools such as Kraken2 [34]. Concerning microbiota gene expression analysis, the structural and functional complexity of annotated genes can be resolved by tools capable of reducing genes, such as Cd-hit [35] and DeepNOG [36], respectively. The structural and functional redundancy reduction allows a lower computational expenditure in protein structure prediction with software like AlphaFold able to elucidate the structure and reactivity of proteins [37]. Understanding protein structures can facilitate a mechanistic understanding of their function. With a broader perspective, multi-omics analysis (a combination of metagenomics, meta-transcriptomics, and metabolomics approach [38]) enables a more precise definition of the molecular network involved in a disease. Furthermore, the various omics datasets could be combined using potent tools such as the Multi-Omics Factor Analysis (MOFA) framework [39], which interprets multi-layer high-dimensional data and infers an interpretable low-dimensional representation in terms of a few latent factors (Fig. 2**.**). This tool has been proven effective in the interpretation and implementation of transcriptomic and metatranscriptomic factors in several gastroenterological esophageal and non-esophageal diseases [19].

**Fig. 3:**
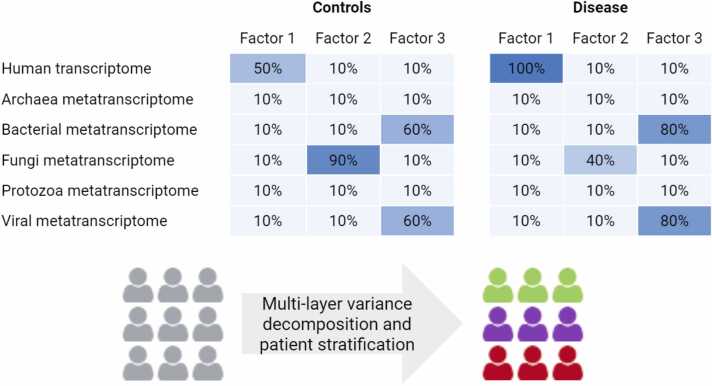
Multi-omic Factor Analysis (MOFA) evidencing a patient stratification according to multi-layer variance.

Notably, by using all these approaches, the scientific community has been generating over the years a broad amount of data from heterogeneous cohorts with similar, but not identical, experimental methods, burdening the simultaneous analysis and comparison among different sources. Considering the huge effort made and the valuable resource these differentiated data may represent, the possibility of combining them in one single analysis would be key to increasing data significance and relevance and, last but not least, the sample sizes. One of the methods to combine data coming from different sources is batch correction before the comprehensive analysis, as recently shown [18], [19], [40] (Fig. 3). This is indeed necessary to remove or maximize the impact of different technical factors, thereby facilitating the identification of significant and relevant biological dissimilarities between samples from different experiments.

In conclusion, the use of molecular and computational techniques has the potential to improve our understanding of the esophageal microbiota and its role in esophageal health. These techniques will strengthen future research aimed at the development of new diagnostic and therapeutic strategies for esophageal disorders in the future.

## 3 Microbiota profiling in benign esophageal diseases

### 3.1 Gastroesophageal reflux disease (GERD) and Barrett's esophagus (BE)

GERD is the most diagnosed esophageal condition, responsible for bothersome symptoms, affecting worldwide patients' quality of life [41], [42]. Concerning epidemiology, GERD has been rapidly spreading especially in Western countries, where 20% of adults results to be affected [20], [43], [44]. GERD usually manifests with reflux symptoms, particularly heartburn and regurgitation, possibly causing the so-called erosive esophagitis [20]. Several factors are involved in GERD occurrence: an increased time of contact between esophageal mucosa and gastroesophageal reflux overcomes the defensive mechanisms of esophageal mucosa (esophageal clearing, salivation, histologic/functional endothelial configuration, and bicarbonate secretion by esophageal submucosal glands) [20], [45]. Macroscopic and microscopic inflammation related to GERD has been classically thought as the result of direct chemical damage of gastric acid and duodenal bile salts [46]. However, recent pieces of evidence have shown that a concomitant immunogenic pathway could be potentially involved [46], even if to date it remains unclear which mechanism first occurs. In fact, it has been demonstrated that, in the distal esophagus of GERD patients, a submucosal (cytokine-induced) inflammation, with intact epithelial cells was found, probably due to an immunoreactivity pathway [47].

GERD is currently differentiated from a clinical perspective into the actual Reflux Esophagitis (RE), in the presence of macroscopically inflamed mucosa, and Non-Erosive Reflux Disease (NERD) [48]. Recently the interest in esophageal microbiome in affecting reflux disease, especially triggering esophageal inflammation and subsequent modifications, has been raised. A recent study by Zhou and colleagues has reported an overall prominent abundance of *Bacteroidetes* in NERD patients versus healthy controls with a slightly higher abundance of *Firmicutes* [49]. Interestingly, in the same study, RE patients with active esophageal distal inflammation, tended to show an esophageal flora with more resemblance to the one of BE and EAC patients [49]. A clear shift from *Bacteroidetes* species to a predominant *Fusobacteria* and *Proteobacteria*-shaped esophageal microbiome in RE compared to NERD could suggest a role of these Gram^-^ bacteria in the pathogenesis of erosive damage and inflammatory cascade [50]. In this setting, lipopolysaccharide (LPS), a cell wall constituent of Gram^-^ bacteria, crucial in maintaining the integrity and viability of bacterial cells, has been studied as involved in the modulation of susceptibility to inflammation in patients with GERD through the interaction with toll-like receptors 4 (TLR-4) and the subsequent activation of the innate immune system [47], as shown in in vitro and in vivo studies [51], [52]. Furthermore, immune system involvement through several inflammatory mediators could explain the relaxation of the lower esophageal sphincter (LES), via interleukin (IL) 18 and other mediators, which in turn stimulates the production of hydrogen peroxide, acting on local smooth muscle and leading to LES relaxation [46], [53]. Of note, *Dorea spp*, of the *Firmicutes phylum*, has been reported higher in GERD patients versus healthy controls, remarking the role of hydrogen-producing species in the pathogenesis of the disease [49].

Of note, the LPS appears to be the most diffuse and known cell wall component of Gram^-^ bacteria (particularly *Fusobacteria* and *Proteobacteria*), and a potential causative factor for microbiome-induced inflammatory cascade related to GERD [51], [54]. In detail, the interaction between LPS and TLR4 due to the disruption of the epithelial barrier induces the activation of an inflammatory cascade mediated by IL18, together with other proinflammatory cytokines such as IL8, IL6, IL1, tumor necrosis factor-alpha (TNFα), and mediators like nitric oxide synthase (NOS) [15], [51]. These molecules favor the relaxation of the LES and promote a delay in gastric emptying mediated by cyclooxygenase 2 (COX2). Accordingly, in the picture of increased abundance of *Fusobacteria and Proteobacteria* in the damaged esophageal mucosa in RE compared to NERD, the presence of *Campylobacter spp*, above all *C. concisus*, was observed at the site of histologic inflammatory and metaplastic changes in GERD and BE patients, together with increased levels of IL18 [51], [55], [56]. Such focal effect of *C. concisus* colonization could explain a local bacterial adhesion facilitated by a biofilm formation, a phenomenon that can be found also in oral and colonic pathology [57]. In response to the mucosal chronic inflammation induced by reflux, intestinal metaplasia of the distal esophageal epithelium with the transition from the healthy stratified squamous epithelium to columnar mucosa occurs, inducing BE [58]. The same inflammatory cascade seen in GERD pathogenesis, mediated by IL1b, IL6, and IL8, is likely to be involved in the transition to metaplasia, as shown in animal models, by acting also on gastric cardiac stem cell progression to dysplasia [59]. As observed in GERD patients, microbiota dysbiosis has been reported also in BE patients [60]. Snider et al. profiled comprehensively eosphageal microbiome of GERD patients (with or without BE) versus healthy subjects, describing two main types: type I microbiome, associated with the healthy condition, displaying a predominance of Gram pos bacteria, like *Streptococcus* genus; while a type II microbiome, associated with RE and BE, characterized as abovementioned by a notable predominance of Gram neg and microaerophiles bacteria, particularly *Fusobacteria* and *Proteobacteria* [60]. Such transition from Gram^+^ to Gram^-^ predominance could be addressed as one of the driver of the metaplastic transition in BE [59]. Concomitantly Lopetuso and colleagues found a decrease in *Bacteroidetes* (particularly *Prevotella spp*) in the metaplastic tissue by comparison with healthy mucosa [61]. It has been argued that the expression of LPS from Gram^-^ bacteria could activate the TLR4-NFkB pathway, thus inducing the proinflammatory cascade and contributing to the transition of healthy mucosa to columnar metaplasia. Furthermore, this phenomenon may be influenced also by gastric acid, by killing esophageal acid-sensitive bacteria [62]. Finally, also colonic microbiome could be involved in this process, since an increased *Firmicutes/Bacteroidetes* ratio, found in BE patients, was proposed to be responsible for fermentable oligo-, di-, monosaccharides, and polyols (FODMAPS) fermentation and Short-Chain Fatty Acid (SCFA) production with decreased LES tone and decreased gastric motility mediated by neuropeptide PYY [63](75)(53). In addition to these considerations, *Helicobacter Pylori*, the main etiological factor of atrophic gastritis and gastric intestinal metaplasia, has been thought related to a defensive role for GERD [64], for its ability to reduce gastric acid secretion, even if it determines a reduction in gastric microbial diversity [65], [66], [67], [68], [69]. Furthermore, the metabolic activity of the colonic microbiota could influence the evolution of GERD, mostly with the production of SCFAs due to the drop in FODMAPS, inducing the relaxation of the LES tone [70], [71]. In conclusion, based on these findings, dysbiosis has been established as a player in the occurrence and progression of several esophageal diseases, including GERD and BE, by the upregulation of inflammatory cascades and changes in microbiome composition. Since other factors could influence the esophageal microbiome, further studies are needed to better elucidate the mechanisms involved in esophageal dysbiosis ultimately unveiling new potential targets for treating these diseases.

### 3.2 Achalasia

#### 3.2.1 Is it a virus-induced disease?

Esophageal achalasia is a motor disorder characterized by the loss of myenteric neurons in the LES and the esophageal body [72]. The etiology of the disease has yet to be clarified, but there is evidence suggesting a potential viral cause [73]. Robertson and colleagues conducted a pioneer serological study measuring antibody titers against *herpes simplex type 1* virus (HSV-1), *cytomegalovirus* (CMV), and varicella-zoster virus (VZV) in achalasia patients [74]. Their findings revealed a significantly higher incidence of VZV antibodies in achalasia patients compared to controls. Subsequently, Facco and colleagues investigated the T-cell receptor repertoire and the antigen recognition ability of LES-infiltrating T lymphocytes specific to HSV-1 in achalasia patients [75]. They observed an increased lymphocytic infiltrate in the esophageal tissue of achalasia patients, primarily consisting of CD3^+^CD8^+^ T cells. Analysis of the TCR beta chain repertoire revealed a restricted diversity of TCR beta variable gene families, suggesting a disease-associated oligoclonal selection of T cells [75]. Moreover, lymphocytes derived from the LES of achalasia patients exhibited enhanced proliferation and release of Th1 cytokines upon exposure to HSV-1 antigens. In a separate investigation, Ikebuchi and colleagues explored the expression profile of microRNAs (miRNAs) in achalasia patients compared to controls [76]. Their analysis identified significant overexpression of HSV-1-derived viral miRNAs, specifically hsv1-miR-H1 and -H18, in achalasia patients [76]. This suggests a potential involvement of neurotropic viral miRNAs in achalasia pathogenesis, claiming further investigation to elucidate their functional significance. Additionally, Naik et al. examined the presence of VZV in the esophagus of achalasia patients [77]. Their study detected salivary VZV DNA in a substantial proportion of the achalasia cohort, along with the identification of transcripts encoding VZV late gene products in surgically excised esophageal tissues [77]. The immunocytochemical analysis confirmed the presence of VZV within esophageal neurons and nerve fibers. These findings lend support to the hypothesis that reactivation of latent VZV in esophageal neurons could contribute to chronic VZV infection, subsequently impairing esophageal motility and sphincter control in achalasia [77], [78]. Furthermore, Furuzawa-Carballeda and colleagues investigated the presence of the SARS-CoV-2 virus and its Acetylcholine Converting Emzyme-2 (ACE2) receptor, as well as the associated immune response, in the LES muscle of achalasia patients with a history of COVID-19 infection [79]. They detected the presence of SARS-CoV-2 in the LES muscle fibers in achalasia patients who had experienced COVID-19 infection, while the virus was absent in healthy controls. ACE2 receptor expression was observed in both patients and controls(83). These findings may suggest a potential impact of SARS-CoV-2 on the myenteric plexus, leading to an imbalance between effector CD4 + T cells and regulatory mechanisms in achalasia patients with a history of COVID-19 infection [80]. In conclusion, several studies have provided evidence supporting a viral etiology for esophageal achalasia, specifically implicating VZV, HSV-1, and SARS-CoV-2 as potential triggers. It is possible to hypothesize that viral infections may contribute to the development of achalasia. However, conflicting results from other studies [81], [82] emphasize the need for further research to establish a definitive causal relationship.

#### 3.2.2 Role of microbiome

Esophageal microbiota in achalasia has been investigated to understand its possible role in the etiopathogenesis of the disease. Pajecki et al. first studied the microbiota in the chagasic megaesophagus (a secondary variant of esophageal achalasia) and compared it to the healthy tissue [83]. They found that patients with megaesophagus showed wide variability in microbiota composition, predominantly composed of aerobic Gram^+^ and anaerobic bacteria. The bacterial concentrations varied according to the degree of esophageal dilation, with higher concentrations observed in patients with more severe megaesophagus [83]. *Streptococcus* was the most common aerobic Gram^+^ genera evaluated, while *Veillonella* was the most frequent anaerobic bacteria. Another study included 29 patients with achalasia who underwent treatment with peroral endoscopic myotomy (POEM) of the lower esophageal sphincter [84]. The study analyzed mucosal biopsies and esophageal fluid retention samples, before and after POEM. The dominant phyla in the esophageal microbiota were *Firmicutes, Bacteroidetes, Proteobacteria, Actinobacteria*, and *Fusobacteria*. *Streptococcus* was by far the most abundant genus. Analysis of alpha and beta diversity revealed significant differences between tissue and fluid samples. However, esophageal microbial composition showed no significant changes 8 weeks post-POEM [84]. A subsequent investigation by Yeh et al. compared achalasia patients to normal controls and observed differences in microbial composition [85]. Patients had a predominant abundance of *Firmicutes, Bacteroidetes*, and *Proteobacteria* at the phylum level, with *Streptococcus, Prevotella, Lactobacillus, Veillonella, Neisseria*, and *Alloprevotella* as prominent genera [85]. The control group showed similar phyla predominance but had different genus distributions. *Lactobacillus, Megasphaera*, and *Bacteroides* resulted as significantly increased in the achalasia population. Moreover, patients with more severe achalasia (such as those with esophageal food retention) had a higher abundance *Lactobacillus*. Twenty patients were analyzed after endoscopic treatment with POEM. The alpha diversity of the esophageal microbiota did not differ before and after treatment. However, beta diversity analysis showed a significant change in the microbial community after POEM, as Neisseria was significantly enriched, while the abundance of *Lactobacillus* and *Bacteroides* significantly decreased [85]. Achalasia patients with erosive esophagitis (55% of the cohort) showed increased alpha diversity and an increase in the genus *Lachnoanaerobaculum*. In conclusion, the role of the esophageal microbiome in understanding the development of achalasia, including its pathogenesis and potential carcinogenesis, seems promising. Achalasia leads to stagnant and slow clearance of the distal esophagus, creating a favorable environment for microbial growth [86]. Certain bacterial taxa like *Lactobacillus* and *Enterobacteriaceae* are enriched in achalasia esophageal tissues and commonly found in EAC as well, while *Veillonella* is increased in GERD and BE [87]. Evidence showed that the esophageal microbiome is modulated depending on the degree of esophageal dilatation, which is greater in the late stage of the disease, and in response to treatment, suggesting the existence of a dynamic bacterial environment. Future studies investigating the cause-effect role of esophageal microbiome in the pathogenesis, cancer risk, and treatment outcomes of achalasia are warranted since the pilot studies on microbiome composition in achalasia mainly focused on describing the microbial environment before and after treatment or based on esophageal anatomical alterations.

### 3.3 Eosinophilic esophagitis: microbiome signature and pathogenic hypothesis

Eosinophilic esophagitis (EoE) is a chronic inflammatory disease characterized by a T-Helper type 2 (Th2) inflammatory response, with food antigens as the main causative factor, resulting in eosinophils accumulation within the esophageal mucosa [88]. A pivotal role in EoE pathogenesis is played by several molecules associated with Th2 response, such as IL5 and IL13 [89]. Nowadays, EoE incidence and prevalence are steadily increasing, being reportedly 3.7/100,000/year and 22.7/100,000 respectively, in the most recent metanalysis [90]. Various risk factors have been associated with EoE pathogenesis, including allergic/atopic conditions and environmental factors [91], [92], genetic predisposition with up to 60% monozygotic twins concordance [93], or even the lack of *H. Pylori* infection [94], [95]. In recent years the possible role of pattern recognition receptors (PRR) in the pathogenesis of EoE has been mainly hypothesized because of the involvement of innate immune system cells in the Th2 inflammation response (mast cells and natural killer cells, among others) [86], [96]. This has raised the concern of a possible involvement of resident esophageal microbiota and its changes as a trigger or sustain of EoE [97]. Benitez and colleagues assessed esophageal core microbiota composition in 33 pediatric EoE patients versus healthy controls, finding an increased abundance of Gram neg bacteria (*Proteobacteria* genus above all) in active EoE compared to healthy subjects, with a huge prevalence of *Firmicutes* phyla (particularly Gram pos bacteria, *Streptococcus* genera above all) in healthy subject-derived biopsies compared to EoE [98]. The findings from this research received further confirmation by another study by Harris et al., who evaluated through *16S rRNA* sequencing the esophageal microbiome in EoE patients, GERD patients, and healthy individuals. Their results confirmed an increased abundance of total bacterial load in EoE and GERD samples compared to the healthy control [99]. A concomitant decrease in Gram+ bacteria and an increase in the Gram^-^ (particularly *Haemophilus influenzae* and *Pasteurella multocida*) were detected in disease versus healthy subjects [99]. Hiremath and colleagues in 2019 analyzed 15 active EoE patients versus 11 inactive EoE and 19 healthy subjects, from oral swabs samples, confirming an increased abundance of *Haemophilus Influenzae* in active EoE, with a coherent slight decrease in *Streptococcacae* and *Lactobacillacae* [100]. In patients under PPI therapy, a slight increase in Gram^+^ bacteria was observed [100]). The latest updated systematic reviews on esophageal microbiota profiling in EoE confirmed these pilot results [22], [101], as shown in Table 1. Being PPIs the first-line treatment for these patients, this is a relevant confounding factor of all studies on esophageal microbiota profiling since the reduction of acid gastric production and reflux intervenes in the esophageal microbiome modulation [102]. The first pieces of evidence describing the impact of conventional therapies on esophageal microbiome composition and diversity have recently been exploited [102]. Particularly, it seems reasonable that dietary treatment (the so-called Food Elimination Diets (FEDs)) could affect the composition of the core esophageal microbiome. In the study by Laserna-Mendieta and colleagues, alpha diversity (intra-sample), measured by Shannon and inverse Simpson indexes, did not result different between pre and post-treatment study groups, while considerably higher compared to the controls [102]. The only significant reduction was detected for Simpson index pre- versus post-treatment in the FED group (p < 0.01). This study added an interesting evaluation of beta diversity (inter samples) through Amplicon Sequence Variants (ASV) with the use of non-metric multidimensional scaling (NMDS) [103] of the Bray–Curtis similarity index [104], a widely known method to describe microbial ecosystems, correlating microbial experimental profiling to pre-defined patterns. The results showed a lower beta diversity for EoE patients who underwent a FED treatment compared to untreated EoE cases [102]. A more recent work by Facchin and colleagues has established how advanced bio-informatics could give us tools to discriminate EoE patients from healthy subjects through microbiota profiling [105]. In this work authors collected salivary samples together with esophageal and gastric biopsies from EoE and non-EoE patients, identifying 20 ASVs able to discriminate EoE from non-EoE subjects with specificity and sensitivity values up to 80% [105]. The use of advanced techniques of computational analysis is a novel field still in development. Through the use of MOFA [106], a recent meta-transcriptomic metanalysis from an Italian group [18] has displayed how esophageal dysbiosis could correlate with several factors of discrimination based on different esophageal microbiome profiles. These results were pooled in the EoE TaMMa web application (https://eoe-meta-analysis.herokuapp.com/), an innovative freely available tool to implement basic and translational research in the field of EoE [18]. Altogether these pieces of evidence point out the role of gut microbiota in the pathogenesis of chronic inflammatory gastrointestinal disease as key and new studies are warranted to explore its involvement in the maintenance of esophageal eosinophilia and inflammatory triggers in EoE.

**Table 1:** Studies about microbiota assessment in eosinophilic esophagitis.

Study (year)	Design	Population	Method	Samples	Outcomes
Benitez et al. 2015	Retrospective	Active EoE 18, inactive EoE 15, HC 35	16S rRNA gene amplification	Esophageal biopsies+saliva	EoE ↑ *Proteobacteria*, *Bacteroidetes*; HC ↑ *Firmicutes*
Harris et al. 2015	Retrospective	Active EoE 11, inactive EoE 26, HC 25, GERD 8	16S rRNA gene amplification and 454 pyrosequencing	Esophageal tissue with EST	EoE ↑average bacterial load; EoE and HC similar composition in *Firmicutes, Proteobacteria*, *Bacteroidetes*; GERD ↑ *Firmicutes* and ↓ *Proteobacteria*
Hiremath et al. 2019	Retrospective	Active EoE 15, inactive EoE 11, HC 19	16S rRNA gene amplification	Salivary samples	EoE ↓ *Leptotrichiaceae*, *Actinomyces*, *Lactobacillus, Streptococcus;* HC ↑ *Neisseriaceae*, *Haemophilus*.
Laserna-Mendieta et al. 2021	Retrospective	Active EoE 30, HC 10	16S rRNA gene amplification	Esophageal biopsies pre-treatment versus post FED, STC, PPI	*Firmicutes* (65%), *Proteobacteria* (18%) and *Bacteroidetes* (9%) [EoE vs HC, p = NS) Post-STC ↓ *Firmicutes* ↑ *Proteobacteria, Bacteroidetes, Fusobacteria*
Facchin et al. 2022	Retrospective	Active EoE 29, HC 20	16S rRNA gene amplification	Salivary samples	EoE ↑ *Streptococcus cristatus, Prevotella oris, Veillonella massiliensis and Peptostreptococcus stomatis Eubacterium nodatum, Porphyromonas, Alloprevotella, Selenomonas* and other *Streptococcus* genera. HC ↑ *Prevotella, Alloprevotella, Porphyromonas, Neisseria* and *Streptococcus* genera, *Mogibacterium,* *Haemophilus pittmaniae*

EoE Eosinophilic Esophagitis; EST Esophageal String Test; FED Food Elimination Diet; GERD Gastroesophageal Reflux Disease; HC Healthy Controls; PPI Proton Pump Inhibitor Therapy; STC Corticosteroid therapy

## 4 Malignant esophageal disease

Recent research has provided evidence that the microbiome may also influence carcinogenesis, tumor progression, and patient outcomes [107], [108], [109]. Microbial colonization of tumor sites could instigate genomic instability via DNA damage induction and impaired cell cycle regulation, thereby promoting the accumulation of oncogenic mutations that drive malignant transformation. Bacteria may modulate cellular signaling pathways through their release of bioactive inflammatory molecules into the tumor microenvironment [110]. Disruption of normal signaling kinetics could facilitate neoplastic evolution and uncontrolled proliferation [110]. Perturbations of the intricate balance between microbes and anti-tumor immunity could precipitate immune evasion by the developing neoplasm and undermine immunological control of nascent cancer cells [134]. EC is the eighth most common cancer and the sixth leading cause of cancer death worldwide [111]. Its development is influenced by chronic inflammation and environmental and genetic factors, but its etiology and pathogenesis are not fully understood yet [112]. Growing evidence shows that the human microbiota plays a vital role in EC carcinogenesis [113]. The two main histological subtypes of EC, esophageal squamous cell carcinoma (ESCC) and EAC account for the majority of cases [111]. Several studies have investigated the esophageal microflora in patients with ESCC. Using *16S rRNA* gene sequencing, Yang et al., characterized differences between ESCC esophagi and healthy individuals [114]. Reduced diversity and lower abundance of the bacterial phyla *Bacteroidetes*, *Spirochetes*, and *Fusobacterium* were found in tumor tissues compared to controls. An index based on these dysbiotic taxa, discriminating ESCC from healthy esophagus was also developed. The ESCC microbiota also exhibited altered nitrate and nitrite reductase activities. Comparable distinctions were detected when comparing esophageal microbiota of ESCC tissue to esophagitis and healthy controls [115]. ESCC patients displayed lower *Faecalibacterium*, *Bacteroides*, and *Blautia* but higher levels of *Streptococcus*. There were also gradual decreases in *Megamonas*, *Collinsella*, *Roseburia*, and *Ruminococcus_2* from controls as compared to esophagitis and ESCC. Using multilevel linear discriminant analysis effect size (LEfSe), increased levels of *Actinobacillus*, *Peptostreptococcus*, *Streptococcus*, *Prevotella*, and *Fusobacterium* were registered. Li et al. investigated alterations in the esophageal microbiota during esophageal carcinogenesis, from healthy esophagus to esophagitis, low grade dysplasia (LGD), high grade dysplasia (HGD), and ESCC [116]. *16S rRNA* gene sequencing of biopsies and swab specimens revealed significant differences in *Neisseria, Haemophilus, Streptococcus*, and *Porphyromonas*. *Streptococcus* decreased from normal to ESCC, while the others increased. Among models based on combinations of characteristic genera, *Streptococcus* and *Neisseria* showed the strongest association with ESCC status and disease progression (area under the curve [AUC] 0.738) and showed potential for discriminating ESCC patients from controls [116]. Another large-scale study investigated salivary and esophageal microbiota in individuals with ESCC and precursors. *16S rRNA* gene amplicon profiling and sequencing revealed microbial diversity decreased with disease progression from normal to LGD to HGD and ESCC. *Granulicatella, Rothia*, *Streptococcus, Gemella, Leptotrichia* and *Schaalia* were enriched in LGD; *Lactobacillus* was enriched in HGD while *Bosea, Solobacterium, Gemella* and *Peptostreptococcus* were enriched in ESCC. In addition, functional predictions identified decreased nitrate reductase functions in ESCC samples [117]. Moreover, esophageal microbiota between patients with ESCC and adjacent non-tumor tissue was compared, by sequencing of *16 S rDNA* from 19 paired tumor and non-tumor samples, revealing differences in composition and diversity. Specifically, the ESCC microbiota showed a characteristic genus-level profile dominated by *Streptococcus* (6.93% of the microbiota) and reduced diversity compared to non-tumor tissue dominated by Labrys (11.1% of the microbiota) [118]. Similarly, Lin et al. analyzed samples from 120 ESCC patients using *16S rRNA* gene sequencing and demonstrated differential abundance of 56 taxa, including enrichment of species like *Rothia mucilaginosa* and *Peptostreptococcus endodontalis* in tumor tissues and reduction of *H. pylori* in adjacent non-tumor tissues, as confirmed by qPCR [119]. Hu et al. unsuccessfully tried to find correlations between the microbiota and ESCC location or stage using *16 S rRNA* gene sequencing; however, morphology and lymph node metastasis seemed to influence the microbiota. Following the metastasis path, *Proteobacteria* abundance decreased while *Bacteroidetes* abundance increased; so even if not associated with location or stage, certain bacterial taxa may play a role in ESCC progression [120]. On the contrary, Zhang et al. found that *Treponema* and *Brevibacillus* were enriched in N1 and N2 stages respectively in ESCC tissues, while *Acinetobacter* was enriched in the T3 stage (meaning a tumor invading peri-colic tissues). In non-tumor tissues, *Fusicatenibacter* was enriched in the T2 stage (muscularis propria invasion), while *Corynebacterium, Aggregatibacter, Saccharimonadaceae-TM7x* and *Cupriavidus* were enriched in the T4 stage (invasion of adjacent organs or visceral peritoneum). Functional analysis also revealed that bacteria involved in nitrotoluene degradation were enriched in non-tumor tissues, while those associated with base excision repair were enriched in tumor tissues [121]. In this context, *Fusobacterium nucleatum* is probably the most studied in ESCC for the role it plays. Different studies showed *F. nucleatum* is more prevalent in metastatic than non-metastatic ESCC and *F. nucleatum*-positive ESCC patients had shorter cancer-specific survival and higher cancer-specific mortality. Additionally, it was closely associated with ESCC pT stage and clinical stage [122], [123]. Instead, according to Kovaleva et al. [124], only bacteria from the *Staphylococcus* genus showed differential abundance between tumor and normal tissues. A significant correlation was found between higher bacterial burden and a pro-tumorigenic phenotype in the ESCC microenvironment, characterized by elevated CD206 ^+^ macrophage expression and inducible nitric oxide synthase (iNOS) levels in tumor cells. Within tumors exhibiting a high proportion of CD206 + macrophages, Gram^+^ bacteria predominated over Gram^-^ and higher Gram^+^ bacterial load correlated with poorer prognosis. Numerous investigations have been conducted to examine alterations in esophageal microbiota in EAC, with mixed data on microbial composition. Some research found EAC patients exhibiting lower microbiome diversity compared to controls [125], [126]; differently, others demonstrated that EB and EAC patients have higher microbiome diversity compared to controls [63]; while certain studies reported no significative differences [127]. Snider et al. observed a notable increase in *Proteobacteria* and reductions in *Firmicutes* and an increase in *Verrucomicrobiaceae* and *Enterobacteriaceae* in esophageal samples of EAC patients [126]. Similarly, another study found a high prevalence of *E. Coli* in EAC tissues, while lacking in the non-tumor adjacent epithelium, confirming the shift toward *Enterobacteriaceae* in esophageal carcinogenesis [128]. Nonetheless, there are discrepancies concerning the possible prevalence of Gram^-^ bacteria in EAC tissue [63], [125]. The study conducted by Elliott et al. found that EAC microbiota was characterized by an abundance of lactic acid-producing bacteria, encompassing *Lactobacillus, Staphylococcus, Bifidobacterium*, and *Streptococcus*. Researchers speculated that *Lactobacillus spp.* dominate the niche due to their ability to resist low pH and inhibit competitor bacteria through lactic acid production [125]. As well established, high lactate levels promote tumorigenesis and progression through angiogenesis, immune escape, cell migration, and metastasis [129]. Additionally, a high prevalence of *Candida albicans* and *Candida glabrata* in EAC samples was found, indicating the existence of esophageal fungal microbiota [128]. The role of *H. pylori* in EAC risk remains unclear. A meta-analysis of 28 studies found an inverse relationship, suggesting that *H. pylori* infection may confer protection against EAC [130]. However, other studies have found no clear association or have produced inconclusive results [131]. Dysbiotic changes in esophageal microbiota during the EAC cascade may promote its pathogenesis through several mechanisms: 1) Increased lactate production for the Warburg effect; 2) increase of iNOS expression and LES relaxation; 3) stimulation of COX-2 expression and delayed gastric emptying; 4) activation of the NLRP3-inflammasome; 5) activation of toll-like receptors (TLRs) [132–137]. Studies evaluating esophageal microbial composition in esophageal cancer are summarized in Table 2. The wide variability of microbiota composition or relative abundances of specific taxa in the tumor environment between different studies represents the real challenge and it could be mainly attributed to the wide heterogeneity and patient-dependency of the tumor environment. The specificity of microbiota composition and signature for every patient could be potentially addressed by targeted therapies.

**Table 2:** Studies about esophageal cancer and microbiota composition.

Study (year)	Design	Population	Method	Samples	Outcomes
Yamamura et al. (2016)	retrospective	325 EC	qPCR	Surgical resections	F. nucleatum is associated with worse outcomes
Elliott et al (2017)	prospective	20 controls, 24 BE, 23 LGD+HGD, 19 EAC	16S rRNA	Cytology brush + mucosal biopsy	↓ ↓ ↓ microbial diversity; ↑ lactic acid bacteria (*Lactobacillus fermentum*) in EAC
Snider et al. (2019)	prospective	16 controls; 14 BE; 6 LGD; 5 HGD; 4 EAC	16S rRNA	Cytology brush + mucosal biopsy	↓ Firmicutes, Veillonella in HGD-EAC ↑ Proteobacteria. Enterobacteriaceae, Akkermansia muciniphila in HGD-EAC
Hu et al. (2020)	prospective	54 ESCC	16S rRNA	Surgical resections	↑ ↑ ↑ Proteus, Fusobacterium, Bacteroides; ↓ Proteobacteria; ↑ Bacteroidetes when lymphnodes metastasis
Li M. et al. (2020)	retrospective	70 controls; 70 ES; 70 LGD; 19 HGD; 7 ESCC	16S rRNA	Cytology brush + mucosal biopsy	↑ Neisseria, Haemophilus, Porphyromonas from normal to ESCC; ↓ Streptococcus from normal to ESCC.
Lopetuso et al. (2020)	prospective	10 controls; 10 BE; 6 EAC	16S rRNA	Mucosal biopsy	↓ ↓ ↓ Streptococcus; ↑Prevotella, Veillonella Leptotrichia in EAC. Leptotrichia is the main distinguishing taxa in EAC.
Peter et al. (2020)	prospective	10 controls; 10 IM; 10 LGD; 10 HGD; 12 EAC	16S rRNA	Mucosal biopsies	↓ ↓ ↓ Siphonobacter, Balneola, Nitrosopumilus, Planctomyces
Zhou et al. (2020)	prospective	16 controls; 11 GERD; 20 RE; 17 BE; 6 EAC	16S rRNA	Cytology brush + mucosal biopsies	↑ ↑ ↑ Lactobacillus, Bifidobacterium, Staphylococcus, Streptococcus
Jiang et al. (2021)	prospective	21 controls; 15 ES; 32 ESCC	16S rRNA	mucosal biopsy + surgical resection	↓ ↓ ↓ Faecalibacterium, Curvibacter, Bacteroides; ↓ Megamonas, Collinsella, Roseburia, Ruminococcus_2 from normal to ESCC
Kovaleva et al. (2021)	retrospective	48 ESCC	16S rRNA	surgical resection	*Staphylococcus* differs between tumors and normal tissues; ↑ CD206 + and iNOS in tumor is associated with Gram+ abundance and worse prognosis
Li Z. et al. (2021 a)	prospective	82 controls; 60 LGD; 64 HGD; 70 ESCC	16S rRNA	Cytology brush + saliva specimens	Bosea, Gemella, Lactobacillus, Solobacterium were main biomarkers in ESCC
Li Z. et al. (2021 b)	retrospective	111 ESCC	16S rRNA + qPCR	tissue wax blocks	↑ ↑ ↑ Actinobacteria, Bacteroidetes, Firmicutes, Bacteroidetes, Fusobacteria, Proteobacteria, Spirochetes. ↑ F. nucleatum was correlated with pT and clinical stages
Yang et al. (2021)	prospective	11 controls; 18 ESCC	16S rRNA	mucosal biopsy or surgical resection	↓ microbial diversity; ↓ Bacteroidetes, Spirochaetes, Fusobacterium
Lin et al. (2022)	retrospective	120 ESCC	16S rRNA	surgical resection	↑ Rhizobium, Sphingomycetes in tumor tissues; ↑ Rhodospirillles in tumor adjacent tissues.
Shen et al. (2022)	prospective	51 ESCC	16S rRNA + qPCR	surgical resection	↑ ↑ ↑ Proteobacteria, Firmicutes, Actinobacteria, Deinococcus-Thermus, Bacteroidetes; ↑ Streptococcus in tumor tissues; ↑ Labrys in adjacent non-tumor tissues
Zhang et al. (2022)	prospective	31 ESCC	16S rRNA	surgical resection	↑ Brevibacillus and Treponema at N1 and N2 stages respectively; ↑ Acinetobacter at T3 stages; ↑ Fusicatenibacter in T2 stages non-tumor tissues; ↑ Corynebacterium, Cupriavidus, Saccharimonadaceae-TM7x, Aggregatibacter in T4 stages non-tumor tissues.

BE Barrett Esophagus; EAC Esophageal Adenocarcinoma; EC Esophageal Cancer; ESCC Esophageal Squamo-cellular Cancer; GERD Gastroesophageal Reflux Disease; HGD High-Grade Dysplasia; IM Intestinal Metaplasia; LGD Low Grade Dysplasia

## 5 Post-surgical complications

The correlation between microbiota and post-esophagectomy complications has gained increasing interest and research in recent years. Esophagectomy is a complex surgical procedure that involves the partial or complete removal of the esophagus. It is primarily performed for the treatment of esophageal cancer, including squamous cell carcinoma or adenocarcinoma (with or without Barrett's esophagus). However, it is also performed for noncancerous conditions when previous attempts to preserve the esophagus have been unsuccessful, such as in cases of end-stage achalasia, strictures, or corrosive ingestion. One of the most concerning complications following esophagectomy is anastomotic leakage, which refers to the breakdown or disruption of the anastomosis between the esophagus and the gastric conduit [107]. Post-esophagectomy leaks can lead to significant morbidity, mortality, and increased hospitalization costs [108], [109]. Advancements in surgical techniques have certainly improved patient outcomes, but the incidence of post-esophagectomy leaks remains high, ranging between 11.4% and 21.2% [110], [111]. Treatment strategies vary depending on the patient’s clinical status and time of presentation and may involve endoscopy, such as Endoscopic Vacuum Therapy (EVT) or esophageal stent placement [112], [113], [114], [115], [116], [117], [118], or surgical reintervention. The high prevalence of anastomotic leaks highlights the need for a better understanding of the factors contributing to these complications and potential strategies for prevention and management. In recent years, the role of microbiota has emerged as a potential influencing factor in post-esophagectomy leaks. Studies conducted in the field of colorectal surgery have provided valuable insights into the potential role of microbiota in the development of anastomotic leaks. Preclinical studies using animal models have shown that certain microorganisms, such as *Pseudomonas Aeruginosa* and *Enterococcus faecalis*, can impact the incidence of anastomotic leaks [119]. In a rat model, the inoculation of *Pseudomonas aeruginosa* after radiotherapy was associated with a higher incidence of colorectal anastomotic leak compared to non-colonized rats (60% vs 0%, p < 0.01) [119]. Similarly, an increased presence of *Enterococcus faecalis*, a commensal organism, has been identified as a causal factor in colorectal anastomotic leak formation [120], [121]. These findings indicate that specific microorganisms within the microbiota can directly impact the healing process and integrity of anastomoses. It is hypothesized that these microorganisms may produce enzymes, such as matrix metalloproteinase-9, or activate inflammatory pathways that contribute to collagen breakdown and impair wound healing [120]. Interestingly, some of these microorganisms, such as *Enterococcus faecalis*, have demonstrated resistance to commonly administered antibiotics during the post-operative period, emphasizing their potential impact on post-esophagectomy complications [120]. Additionally, the administration of morphine, a medication commonly used in the postoperative period, has been found to enhance the adhesiveness and collagenase production of high-collagenase-producing strains [122]. While the evidence from colorectal surgery is intriguing, there is still a scarcity of data specifically focused on the correlation between microbiota and anastomotic leaks following esophagectomy. A study conducted by Reddi et al. aimed to investigate this link by analyzing the composition of perioperative esophageal microbiota and the occurrence of post-esophagectomy complications. The study included 55 patients who underwent trans-hiatal esophagectomy for various indications, with the majority having adenocarcinoma or squamous cell carcinoma (44 out of 51 patients). Among these patients, 10 (18%) experienced anastomotic leakage [123]. The researchers found a significant difference in microbiota composition between preoperative saliva samples and intraoperative gastric mucosa samples in patients who experienced anastomotic leakage. This suggests that changes in the microbiota may be associated with the development of anastomotic leaks. However, no direct association between specific microbiota patterns and anastomotic leakage was identified [123]. In a retrospective study involving 783 patients undergoing oncologic esophagectomy, a fecal sample that tested positive for *Proteus mirabilis* was associated with post-esophagectomy pneumonia [124]. This bacterium is known to be linked to latent enteric inflammation, which can lead to systemic inflammation and pose a significant risk of post-surgical dehiscence. Delayed gastric emptying (DGE) is a common complication following Ivor-Lewis esophagectomy, observed in 15–18% of patients [125], [126]. In the short term, DGE can result in complications such as anastomotic leak and pneumonia [127], while in the long term, it is strongly associated to nutritional issues [128]. DGE primarily arises from anatomical and physiological post-surgical abnormalities, such as peristalsis dysfunction resulting from complete vagotomy, gastric conduit torsion [129], and pyloric spasm. However, there is evidence demonstrating that the composition of the microbiota can influence gastric emptying [130]. Furthermore, it is widely recognized that changes in intestinal bacteria can significantly affect gastrointestinal motility [131]. Currently, there is a lack of data, and the relationship between dysbiosis and post-esophagectomy DGE has not been investigated. Further studies utilizing advanced DNA-based microbiota analysis techniques will be beneficial in understanding whether the microbiota plays a role in the pathophysiology of this post-surgical complication.

## 6 Conclusions and future directions

Gut microbiota has been one of the main gastrointestinal research topics in the last 3 decades. The complex interplay between the thousands of microbes forming the gut microbiota environment and our immune and gastrointestinal systems seems pivotal in the comprehension of several diseases' pathogenesis [132]. If more has been studied concerning small-intestinal and colonic microbiota, little is known about the composition and the diversity of microbial environment inhabiting the upper-GI tract, especially in the esophagus [20]. In recent years the initial belief considering the esophagus as a sterile muscular tube with only functions of food trespassing has left room for the detection of a core esophageal microbial signature with relevant interdependence with oral and gastric microbiota composition. *Firmicutes* seem the most abundant phyla adherent on the esophageal mucosa, composed mainly of *Streptococcus* genus together with a high prevalence of *Haemophylus* and *Klebsiella* [98]. A consistent population of commensals viruses and fungi has been evaluated in recent works, paving the way to the interest in the esophageal “virome” and “mycome” aside from the known “bacteriome” [18]. The study of the overall microbial environment, including that of the the esophagus, has been accelerated thanks to the advent of high-throughput sequencing and bioinformatics, able to elaborate thousands and thousands of raw data implementing bio-repositories databanks, and expanding the “multi-omic dimension” knowledge, including metagenomic, meta-transcriptomics, metabolomics and proteomics [39]. Thanks to biomolecular and bioinformatics efforts, microbiota's role in the development of GERD and BE has been strongly suspected. The role of LPS as a probable trigger of the immune system via the TLR4-IL-8 pathway together with the prevalence of Gram ^-^/microaerophiles microbiome in GERD/BE patients compared to HC, seems promising [15]. Peculiar microbiome profiling found in rare idiopathic esophageal disease, achalasia above all, suggests a possible etiopathogenetic factor of specific genera (*Lactobacillus* and *Bacteroides* especially) in the disease development [85].

Finally, the etiopathogenesis of chronic inflammatory disease of the GI tract could possibly be affected by microbiome composition. Recent insights on esophageal microbial composition different in active EoE, compared to inactive disease and HC, have been registered [133]. Last but not least, esophageal microbial environment has been evaluated in esophageal cancer with increasing evidence of a reduction in microbial diversity together with a firm decrease in microbes genera predominant in HC *Fusobacterium* and *Streptococcus* above all) [16]. All this evidence considered, the role of esophageal microbiota composition in various esophageal diseases seems of great interest, and further studies and subsequent implementation of the latest biomolecular and bioinformatics techniques are warranted.

## Funding

No Funding.

## CRediT authorship contribution statement

AB and LM conceived the article. AB, LM, FVM, EV, ES, OL, and EF wrote the article. AB and LM created tables and figures. FU, SC, SS, and SD critically reviewed the content of the paper and supervised the project. The manuscript was approved by all authors.

## Declaration of Competing Interest

Professor Silvio Danese declares the following conflicts of interest: has served as a speaker, consultant, and advisory board member for Schering-Plough, AbbVie, Actelion, Alphawasserman, AstraZeneca, Cellerix, Cosmo Pharmaceuticals, Ferring, Genentech, Grunenthal, Johnson and Johnson, Millenium Takeda, MSD, Nikkiso Europe GmbH, Novo Nordisk, Nycomed, Pfizer, Pharmacosmos, UCB Pharma and Vifor. The other authors declare no conflicts of interest.

A.Barchi, L. Massimino, F.V. Mandarino, E. Vespa, O. Almolla, S. Passaretti, E. Sinagra, T.L. Parigi, S. Cagliani, S. Spanò, F. Ungaro declares no conflict of interest. S Danese has served as a speaker, consultant, and advisory board member for Schering-Plough, AbbVie, Actelion, Alphawasserman, AstraZeneca, Cellerix, Cosmo Pharmaceuticals, Ferring, Genentech, Grunenthal, Johnson and Johnson, Millenium Takeda, MSD, Nikkiso Europe GmbH, Novo Nordisk, Nycomed, Pfizer, Pharmacosmos, UCB Pharma and Vifor.

## Data Availability

No new data were generated or analyzed in support of this research.
